# Hearing loss in Behçet’s syndrome: prevalence and associated factors in a nationwide cross-sectional analysis

**DOI:** 10.55730/1300-0144.6339

**Published:** 2026-06-02

**Authors:** Alper DOĞANCI, Serdar Can GÜVEN, Nuray YILMAZ ÇAKMAK, Nurhan GÜVEN, Salih BAŞER, Neslihan ÖZTÜRK, Şuayip BİRİNCİ, Naim ATA

**Affiliations:** 1 Division of Rheumatology, Ankara Atatürk Sanatory Education and Research Hospital, Ankara, Turkiye; 2 Division of Rheumatology, Ankara Bilkent City Hospital, Ankara, Turkiye; 3 Department of Internal Medicine, Faculty of Medicine, Ankara Yıldırım Beyazıt University, Ankara, Turkiye; 4 Division of Rheumatology, Yıldırım Beyazıt University Yenimahalle Education and Research Hospital, Ankara, Turkiye; 5 Republic of Türkiye Ministry of Health Strategy Development Department, Ankara, Turkiye; 6 Deputy Minister, Ministry of Health of the Republic of Türkiye, Ankara, Turkiye

**Keywords:** Behçet’s syndrome, auditory involvement, hearing loss, vasculitis

## Abstract

**Background/aim:**

The aim of this study was to investigate the prevalence of hearing loss in Behçet’s syndrome and its associations with other disease manifestations, comorbidities, and treatment agents. To the best of our knowledge, this is the most comprehensive study to date addressing this issue.

**Materials and methods:**

The study was designed as a cross-sectional, nationwide study. The Turkish Ministry of Health National Electronic Database (e-Nabız) was used under the supervision of the Ministry of Health to extract patient data. A nationwide cohort was established from patients who had been assigned an ICD-10 code for BS (M35.2) on at least two occasions, separated by a minimum of 3 months, between 1 January 2016, and 31 December 2022, and were therefore considered to have BS. Data on demographics, comorbidities, major disease manifestations other than mucocutaneous and musculoskeletal involvement, and treatment agents ever used for BS were recorded. Within this cohort, patients who had been assigned an ICD-10 code for hearing loss (H90 or H91 and their subcategories) were classified as BS patients with hearing loss. Demographics, clinical characteristics and treatment history were compared between BS patients with and without hearing loss.

**Results:**

A total of 84,240 patients with BS were identified and included in the analysis. Among these patients, 976 were identified as having hearing loss; 487 had sensorineural hearing loss, 71 had conductive hearing loss, 46 had mixed hearing loss, and 464 had unspecified hearing loss. In multivariable analysis, none of the BS-related manifestations remained independently associated with hearing loss. However, male sex, age >50 years at the time of analysis, hypertension, coronary artery disease, chronic kidney disease, malignancy, depression, viral hepatitis, asthma, osteoporosis, and treatment with certolizumab pegol were independently associated with an increased risk of hearing loss.

**Conclusion:**

In this nationwide study, a prevalence of hearing loss of 1.16% was observed among patients with BS. Our findings suggest that older age and the presence of certain comorbidities, rather than BS-specific manifestations, were associated with hearing loss.

## 1. Introduction

Behçet’s syndrome (BS) is a variable-vessel systemic vasculitis that can affect both the arterial and venous systems, regardless of vessel size. The hallmark manifestations are recurrent oral and genital ulcers, which may be accompanied by ocular, musculoskeletal, vascular, neurologic, gastrointestinal, and cutaneous involvement. The etiopathogenesis is multifactorial and involves both genetic and environmental factors; however, it has not yet been fully elucidated. Although prevalence rates vary worldwide, the disease is more prevalent in East Asia and the Middle East. The symptoms of the disease typically begin in early adulthood and can significantly impact the quality of life of affected individuals [1].

Hearing loss has been reported in several forms of systemic vasculitis. Inflammation involving the auditory nerve, middle ear, Eustachian tube, and associated central nervous system structures has been proposed as a potential pathogenic mechanism, resulting in conductive, sensorineural, or mixed hearing loss [2]. In most forms of vasculitis, the prevalence, subtype distribution, and underlying mechanisms of hearing loss have not yet been fully elucidated.

The occurrence of hearing loss in BS has been investigated in several studies, suggesting that a substantial proportion of patients may be affected to varying degrees, predominantly with sensorineural hearing loss [3–11]. Although these studies involved relatively small sample sizes and provided valuable preliminary insights, the prevalence of hearing loss, its associations with disease manifestations and patient characteristics, and its potential relationship with treatment agents remain insufficiently characterized in BS.

In this nationwide cross-sectional study, we investigated the prevalence of hearing loss in patients with BS and its associations with disease manifestations, comorbidities, and treatment agents. To the best of our knowledge, this is the most comprehensive study to date addressing this issue.

## 2. Materials and methods

### 2.1. Study design

The study was designed as a cross-sectional, nationwide study. The Turkish Ministry of Health National Electronic Database (e-Nabız) was used under the supervision of the Ministry of Health to extract patient data. The e-Nabız system was established by the Ministry of Health in 2015 as a national health information system accessible only to authorized individuals and institutions. The system has extensive coverage and encompasses the entire country. The e-Nabız system contains the clinical records of more than 80 million individuals in Türkiye, including demographic information, recorded ICD codes, laboratory results, medication histories, and comorbidities. The Ministry of Health delivers healthcare information services using Big Data technologies, and e-Nabız is integrated with the National Healthcare Information System (NHIS). Through these integrated systems, patient data recorded from 1 January 2016, onward can be accessed.

A nationwide cohort was established from patients who had been assigned an ICD-10 code for BS (M35.2) on at least two occasions separated by a minimum of 3 months between 1 January 2016, and 31 December 2022, and were therefore considered to have BS. Data on demographics, comorbidities, major disease manifestations other than mucocutaneous and musculoskeletal involvement, and treatment agents ever used for BS were recorded. Within this cohort, patients who had been assigned an ICD-10 code for hearing loss (H90 or H91 and their subcategories) were classified as BS patients with hearing loss.

### 2.2. Statistical analysis

Demographics, clinical characteristics and treatment history were compared between BS patients with and without hearing loss. Data were analyzed using SPSS version 22 (IBM Corp., Armonk, NY, USA). Continuous variables were presented as either mean ± standard deviation (SD) or median (interquartile range) values, whereas categorical variables were presented as frequencies and percentages. Categorical variables were compared using the chi-square test, whereas continuous variables were compared using the Student’s t-test. Binary logistic regression analysis was performed to evaluate the independent effects of variables that differed significantly between groups, and odds ratios (ORs) with 95% confidence intervals (CIs) were reported. The Hosmer–Lemeshow test was used to assess the goodness-of-fit of the logistic regression model. A p < 0.05 was considered statistically significant.

### 2.3. Ethical approval

This study was conducted with the approval of the Ministry of Health (approval no. 95741342–020). The study was conducted in accordance with the ethical principles of the Declaration of Helsinki.

## 3. Results

A total of 84,240 patients with BS were identified and included in the analysis. Among these patients, 976 had an ICD-10 code recorded for hearing loss. Of these patients, 487 had sensorineural hearing loss, 71 had conductive hearing loss, and 46 had mixed hearing loss according to the recorded ICD-10 codes. A total of 464 patients had an ICD-10 code for unspecified hearing loss.

Age at the time of analysis (mean ± SD: 54.76 ± 15.34 versus 45.81 ± 15.09 years; p < 0.001) and age at initial admission (mean ± SD: 49.87 ± 15.19 versus 41.07 ± 14.83 years; p < 0.001) were significantly higher in patients with hearing loss. Female sex was less frequent among patients with hearing loss (n [%]: 483 [49.5%] versus 45,269 [54.4%]; p = 0.002). Overall mortality was similar between the groups (n [%]: 29 [3.0%] versus 2644 [3.2%]; p = 0.718) (Table 1).

The frequencies of evaluable BS-related manifestations were generally similar between the groups, except for gastrointestinal involvement (n [%]: 102 [10.5%] versus 6039 [7.3%]; p < 0.001) and demyelinating central nervous system disease (n [%]: 16 [1.6%] versus 786 [0.9%]; p < 0.026) (Table 1). Regarding comorbidities, hypertension, diabetes mellitus, hyperlipidemia, coronary artery disease, other cardiac diseases, cerebrovascular events, malignancy, tuberculosis, viral hepatitis, primary biliary cirrhosis, chronic liver disease, chronic kidney disease, autoimmune thyroid disease, depression, fibromyalgia, asthma, chronic obstructive pulmonary disease, peripheral neuropathy, dementia, and osteoporosis were all significantly more frequent in patients with BS and hearing loss (Table 1). Patients with BS and hearing loss were more frequently treated with certolizumab pegol and tocilizumab (Table 1).

Multivariable analysis including variables that differed significantly between the groups demonstrated that none of the BS-related manifestations remained independently associated with hearing loss. However, male sex, age >50 years at the time of analysis, hypertension, coronary artery disease, chronic kidney disease, malignancy, depression, viral hepatitis, asthma, osteoporosis, and treatment with certolizumab pegol were independently associated with an increased risk of hearing loss in BS (Table 2).

## 4. Discussion

In this nationwide study, a prevalence of hearing loss of 1.16% was observed among patients with BS. None of the BS-related manifestations remained independently associated with hearing loss. However, older age, male sex, and certain comorbidities were independently associated with an increased risk of hearing loss in BS.

Several studies have investigated auditory function in patients with BS. Karadağ et al. reported an increased prevalence of sensorineural hearing impairment in patients with BS compared with controls, which was associated with age and disease duration but not with BS manifestations [3]. In another study involving 27 patients with BS, nearly 60% exhibited some degree of sensorineural hearing loss, without any association with demographic or clinical characteristics [4]. Ak et al. reported similar findings, demonstrating that 55% of patients with BS had sensorineural hearing loss and that age was the only variable significantly associated with this finding [5]. Similarly, pure-tone audiometry identified sensorineural hearing loss in 10 of 29 patients with BS (34.5%) [8]. Although these studies had relatively small sample sizes, they suggested that 34%–60% of patients with BS exhibited sensorineural hearing impairment. The substantially lower prevalence observed in our study is likely attributable to the fact that only clinically recognized cases resulting in the assignment of an ICD code could be captured by our methodology. The identification of hearing loss based solely on ICD codes should be interpreted with caution because the validity and reliability of these diagnoses cannot be confirmed in the absence of objective audiological assessments, and subclinical abnormalities cannot be detected using this methodology. This is likely the primary reason for the discrepancy in prevalence estimates reported across previous studies. Furthermore, there was a serious possibility of classification bias regarding the type of hearing loss. Nevertheless, this may also represent a strength of the study, as the identified cases likely reflect clinically relevant hearing loss that prompted medical evaluation. Therefore, our findings may primarily reflect clinically recognized hearing loss, whereas previous studies often identified subclinical abnormalities through systematic audiological testing. However, the clinical significance of these subclinical auditory abnormalities remains to be fully elucidated. In addition, our findings are consistent with the limited available literature suggesting that age is more strongly associated with hearing loss than BS manifestations. As expected, hearing function declines with age; however, the mean age of patients with hearing loss in our cohort was not markedly advanced, remaining below 60 years despite a broad age distribution. Accordingly, although age appears to play an important role, it should not be considered the sole determinant of hearing loss risk in patients with BS.

Previous studies have suggested that hearing loss may be associated with mucocutaneous and articular manifestations; however, this association could not be evaluated in our study because these manifestations were not assessed [12]. Another study reported a higher frequency of human leukocyte antigen B57 positivity among patients with BS and sensorineural hearing loss [13]. Yet, these findings should be confirmed in larger study groups. Additionally, consistent with previous reports, sensorineural hearing loss was the most common subtype among patients with hearing loss in our cohort. Although this observation may suggest a potential relationship between BS and hearing loss, definitive conclusions regarding causality cannot be drawn from the available data.

To date, the literature has not consistently demonstrated a significant association between sex and hearing loss in patients with BS. Our findings indicated that male sex was independently associated with an increased risk of hearing loss in BS. It is well established that BS tends to follow a more severe clinical course in male patients [1]. The higher frequency of male patients in the hearing loss group may suggest a potential association between hearing loss and a more severe disease course.

Several comorbidities commonly associated with aging were also found to be associated with hearing loss in BS. This association may, at least in part, be attributable to the aging process itself. However, some comorbidities remained independently associated with hearing loss after adjustment for age, which may reflect the overall disease burden and the effects of treatments administered for these conditions. This issue warrants further investigation.

Behçet’s syndrome is primarily treated with systemic glucocorticoids and conventional immunosuppressive or immunomodulatory agents according to disease severity, the presence of organ- or life-threatening manifestations, and refractoriness to conventional therapy [14]. At present, no specific treatment strategy for hearing loss in BS is recommended by the available literature. Furthermore, it is often difficult to determine whether hearing loss represents a manifestation of BS or an unrelated coincidental finding. Nevertheless, several case reports have suggested that tumor necrosis factor-alpha inhibitors may represent a therapeutic option in such clinical scenarios. Anecdotal evidence suggests that adalimumab and infliximab may be effective in the treatment of sensorineural hearing loss in BS [15,16]. Cyclosporine A has also been proposed as a treatment option based on limited clinical experience [17]. Our results did not demonstrate a significant association with any specific treatment agent, except for certolizumab pegol. Given the observational nature of the study, the underlying explanation for this finding cannot be determined, and any interpretation would remain speculative. This issue remains to be elucidated.

This study has several important limitations. First, this was a database-based study and is therefore subject to the inherent limitations of large-scale administrative data analyses. These limitations include the lack of longitudinal disease activity data, the inability to determine disease duration based on symptom onset, and the absence of detailed information regarding immunosuppressive drug exposure, including treatment timing, duration, and cumulative dose. Second, complete otolaryngology records and objective audiological test results were unavailable for review. Furthermore, potential confounding factors that may contribute to hearing loss, including trauma, infections, concomitant medications, congenital conditions, and otosclerosis, could not be evaluated. Therefore, the design of this study does not allow differentiation between hearing loss as a manifestation of BS, a complication of the disease, or a coincidental finding, and no causal relationship can be established. Accordingly, the findings of this study should be interpreted with caution.

Despite these limitations, this nationwide study provides valuable data regarding hearing loss in BS and includes the largest cohort reported in the literature to date. Our findings suggest that older age and the presence of certain comorbidities, rather than BS-specific manifestations, were associated with hearing loss; however, causality cannot be established because of the cross-sectional study design. Further studies are needed to clarify this issue and to better define the clinical characteristics and therapeutic implications of hearing loss in BS.

## Figures and Tables

**Table 1:** Characteristics of patients with Behçet’s syndrome with and without hearing loss.

	BS with hearing loss (N = 976)	BS without hearing loss (N = 83,264)	p
**Age, years, ± SD**	54.8 ± 15.3	45.8 ± 15.1	<0.001
**Age > 50 years, n (%)**	600 (61.5)	30,569 (36.7)	<0.001
**Age at initial admission, years, ± SD**	49.9 ± 15.2	41.07 ± 14.8	<0.001
**Age at final admission, years, ± SD**	53.4 ± 15.2	44.28 ± 15.9	<0.001
**Follow-up duration, days ± SD**	1316.08 ± 779.1	1193.07 ± 766.9	<0.001
**Sex, female, n (%)**	483 (49.5)	45,269 (54.4)	0.002
**Mortality, n (%)**	29 (3.0)	2644 (3.2)	0.718
**BS manifestations other than musculoskeletal and mucocutaneous, n (%)**			
Uveitis	135 (13.8)	10,356 (12.4)	0.190
Arterial aneurysm	7 (0.7)	395 (0.5)	0.274
Pulmonary thromboembolism	10 (1.0)	550 (0.7)	0.164
Thrombosis other than PTE	51 (5.2)	3583 (4.3)	0.159
Inflammatory bowel disease	102 (10.5)	6039 (7.3)	<0.001
Demyelinating CNS disease	16 (1.6)	786 (0.9)	0.026
**Comorbidities, n (%)**			
Hypertension	528 (54.1)	24870 (29.9)	<0.001
DM	254 (26.0)	11,889 (14.3)	<0.001
Hyperlipidemia	181 (18.5)	7544 (9.1)	<0.001
CAD	258 (26.4)	9368 (11.3)	<0.001
Cardiac disease other than CAD	79 (8.1)	3419 (4.1)	<0.001
CVE	111 (5.4)	4320 (5.2)	<0.001
PHT	2 (0.2)	91 (0.1)	0.371
Malignancy	70 (7.2)	2915 (3.5)	<0.001
Tuberculosis	4 (0.4)	130 (0.2)	0.048
Hepatitis B and C	35 (3.6)	1174 (1.4)	<0.001
Primary biliary cirrhosis	66 (6.8)	3764 (4.5)	0.002
Chronic liver disease	7 (0.7)	157 (0.2)	<0.001
Autoimmune hepatitis	1 (0.1)	61 (0.1)	0.738
CKD	47 (4.8)	1515 (1.8)	<0.001
Urolithiasis	29 (3.0)	1840 (2.2)	0.108
Pernicious anemia	1 (0.1)	82 (0.1)	0.969
Autoimmune thyroid disease	13 (1.3)	568 (0.7)	0.015
ITP	0	47 (0.1)	0.458
Depression	374 (38.3)	19,234 (23.1)	<0.001
Fibromyalgia	66 (6.8)	3764 (4.5)	0.001
Migraine	34 (3.5)	2204 (2.6)	0.106
Asthma	164 (16.8)	6846 (8.2)	<0.001
COPD	71 (7.3)	2396 (2.9)	<0.001
Peripheral neuropathy	72 (7.4)	2870 (3.4)	<0.001
Dementia	16 (1.6)	441 (0.5)	<0.001
Glomerulonephritis	1 (0.1)	19 (0.0)	0.108
Osteoporosis	128 (13.1)	5455 (6.6)	<0.001
**Treatment agents ever used for BS, n (%)**			
Cyclophosphamide	5 (0.5)	234 (0.3)	0.177
MMF and MPA	20 (2.0)	1067 (1.3)	0.035
AZA	259 (26.5)	23,686 (28.4)	0.188
CsA	9 (0.9)	535 (0.6)	0.278
Interferon	6 (0.6)	302 (0.4)	0.195
Colchicine	811 (83.1)	69,234 (83.1)	0.963
IFX	33 (3.4)	2612 (3.1)	0.664
ADA	31 (3.2)	2722 (3.3)	0.871
ETN	9 (0.9)	535 (0.6)	0.278
GOL	3 (0.3)	272 (0.2)	0.916
CTZ	12 (1.2)	469 (0.6)	0.006
TCZ	7 (0.7)	210 (0.3)	0.004

BS: Behçet’s syndrome; PTE: pulmonary thromboembolism; CNS: central nervous system; DM: diabetes mellitus; CAD: coronary artery disease; CVE: cerebrovascular event; PHT: pulmonary hypertension; CKD: chronic kidney disease; ITP: immune thrombocytopenic purpura; COPD: chronic obstructive pulmonary disease; MMF: mycophenolate mofetil; MPA: mycophenolic acid; AZA: azathioprine; CsA: cyclosporine A; IFX: infliximab; ADA: adalimumab; ETN: etanercept; GOL: golimumab; CTZ: certolizumab pegol; TCZ: tocilizumab.

**Table 2:** Multivariable logistic regression analysis of factors associated with hearing loss in Behçet’s syndrome.

	OR	95% [CI]	p
**Age > 50 years**	1.69	1.45–1.99	<0.001
**Sex, male**	1.50	1.31–1.72	<0.001
**Hypertension**	1.49	1.27–1.76	<0.001
**CAD**	1.40	1.18–1.66	<0.001
**CKD**	1.40	1.03–1.94	0.031
**Malignancy**	1.34	1.04–1.73	0.022
**Depression**	1.68	1.46–1.93	<0.001
**Hepatitis B and C**	1.99	1.41–2.83	<0.001
**Asthma**	1.64	1.37–1.95	<0.001
**Osteoporosis**	1.34	1.09–1.64	0.004
**CTZ**	2.05	1.14–3.69	0.017

OR: odds ratio; CI: confidence interval; CAD: coronary artery disease; CKD: chronic kidney disease; CTZ: certolizumab pegol.

## Data Availability

The data supporting the findings of this study are maintained by the General Directorate of Information Technologies of the Ministry of Health of the Republic of Türkiye.
